# Age and sex influence the response in lipid metabolism of dehydrated Wistar rats

**DOI:** 10.1038/s41598-022-11587-w

**Published:** 2022-06-02

**Authors:** S. Quirós Cognuck, W. L. Reis, M. S. Silva, S. V. Zorro, G. Almeida-Pereira, L. K. Debarba, L. L. K. Elias, J. Antunes-Rodrigues

**Affiliations:** 1 Physiology Department, Ribeirao Preto Medicine School, University of Sao Paulo, Ribeirao Preto, Sao Paulo, 14049-900 Brazil; 2 Department of Physiological Science, Center of Biological Sciences, Federal University of Santa Catarina, Florianópolis, SC 88040-900 Brazil; 3 Medical Clinic Department, Ribeirao Preto Medicine School, University of Sao Paulo, Ribeirao Preto, Sao Paulo, 14048-900 Brazil

**Keywords:** Ageing, Fat metabolism

## Abstract

Aging is associated a decrease in thirst sensation, which makes old people more susceptible to dehydration. Dehydration produces energy metabolism alterations. Our objective was to determinate the effect of water deprivation (WD) in the lipid metabolism of old male and female rats. Here we show that in the state of WD, aging and sex alters retroperitoneal white adipose tissue (R-WAT) weight of rats, WD old female rats had more lipolysis products than old male rats, a sexual dimorphism in the hormonal response related with metabolism of the adipose tissue of old rats during WD, the expression of P-para mRNA in R-WAT did not present any alteration in animals submitted to WD, the expression of Aqp7 mRNA in R-WAT is altered by WD, age, and sex. Also, WD stimulated an increase in the plasma concentration of oxytocin and the expression of mRNA of the oxytocin receptors in R-WAT.

## Introduction

Aging is associated with decrease in thirst sensation^1^. Hyperosmolarity and dehydration alter energetic metabolism^2^. Thus, hypertonicity condition with mannitol decrease plasma free fatty acids (FFA) in vivo and decreased insulin release in vitro^2^. Additionally, in male Sprague-Dawley rats deprived of water and food for 24, 48, and 72 h showed more loss of body weight and less increase of plasma FFA than fasted rats, but no differences in blood glucose levels and insulin were observed^2^. In addition, Levy and Stevens observed that plasma hyperosmolality stimulates leptin secretion acutely, which happen by a vasopressin-adrenal mechanism^3^.

During dehydration, the neurohypophyseal hormones, vasopressin, and oxytocin (OT) are released. The increase of these hormones helps to kept hydromineral homeostasis^4^. However, OT has been associated with metabolic effects and weight loss in diet-induced obese animals by reducing food intake and visceral fat mass^5,6^. Chronic central OT infusion increases adipose tissue lipolysis and fatty acid β-oxidation but reduces glucose intolerance and insulin resistance^7^. Furthermore, OT increases the expression of stearoyl-coenzyme A desaturase 1 and *N*-oleoyl-phosphatidyl-ethanolamine which are oleoylethanolamide productor, a known PPAR-alpha activator^7^. Furthermore, in young healthy men, OT reduces reward-related energy intake and glucoregulatory response to food intake^8^.

OT has a dual action mechanism on metabolism^9^. OT concentration below 10^–8^ M, which is basal plasma OT level, stimulates glucose incorporation into glycogen, promotes H_2_O_2_ production, and inhibits hormone-stimulated lipolysis. However, OT concentration higher than 10^–8^ M activates glycogenolysis and glucose transport system, which is additive to insulin effect, suggesting different mechanism of action for two hormones. Moreover, the inhibition of hormone-stimulated lipolysis is diminished, thus OT itself becomes lipolytic^9^.

Adipocytes are a major source of glycerol, which is a substrate for hepatic gluconeogenesis^10^. It was showed that OT increased glycerol release in ex vivo incubated epididymal fad pads^7^. Aquaporin subtypes 7 and 9 (Aqp7 and Aqp9) are aquaglyceroporin, transporting water and glycerol, which are found in the plasma membrane of adipocytes and hepatocytes, respectively. The coordinated regulation of both aquaporins leads to a balance between the release of glycerol by adipocytes and its uptake by the liver^10^.

There are some evidences about sex-specific differences in adipose tissue energy metabolism, such as non-oxidative FFA clearance is higher in women than in men, catecholamine-induced rate of FFA mobilization from visceral fat to the portal venous system is higher in men than women, the women have the highest rate of triglycerides (TG) synthesis than men, circulating levels of the hormone leptin are increased in women compared to men, whereas male mice show higher leptin concentrations compared to female mice, and adiponectin levels are higher in females than in males^11–13^.

Although dehydration is known to produce energy metabolism alterations, little information is available about this issue, and the hormonal and lipid metabolic response to dehydration of elderly and old animals, moreover sex-specific differences have not been described. Thus, our objective was to determinate the effect of dehydration in the lipid metabolism of old male and female rats. We hypothesize that dehydration can trigger hormonal changes that alter the lipid metabolism of old animals, and that this response to osmotic stimulation is different between males and females.

## Results

### Males

#### Body and retroperitoneal white adipose tissue (R-WAT) weight

Table 1 shows that 18-month-old rats had higher body weight than 3-month-old rats, the 3-month-old rats submitted to water deprivation (WD) showed lower body weight than respective control, and the body weight change was bigger in males submitted to WD than control group. The R-WAT was higher in the 18-month-old rats than 3-month-old rats. The 18-month-old WD males showed lower R-WAT weight than the respective control (Table 1).

**Table 1:** Body weight (BW), body weight change, and R-WAT of 3- and 18-month-old control and male and female rats with water deprivation (WD).

Parameter	Males				Females			
	3 months		18 months		3 months		18 months	
	Control	WD	Control	WD	Control	WD	Control	WD
BW (g)	566.8 (38)^ac^	476 (49.4)^bc^	810.1 (147.9)^a^	733 (123)^b^	385.8 (18.4)^ac^	332.8 (20.2)^bc^	522.2 (71.7)^a^	466.8 (59.4)^b^
BW Change (g)	8.9 (28.6)^c^	− 79.7 (6.7)^c^	− 8.6 (9.0)^c^	− 83.2 (15.5)^c^	− 3.9 (12.8)^c^	− 54.1 (5.0)^c^	− 7.0 (11.3)^c^	− 51.5 (5.4)^c^
R-WAT (g/100gbw)	0.78 (0.21)^a^	1.10 (0.44)^b^	2.27 (0.74)^ac^	1.68 (0.73)^bc^	1.18 (0.20)	1.12 (0.42)^b^	1.68 (0.52)	2.22 (0.60)^b^
n	9	10	8	8	11	13	6	6

Values are expressed as means (SD).

^a^Different between age in the control condition p < 0.05.

^b^Different between age in the rats with water deprivation (WD) p < 0.05.

^c^Different between control condition in the same age p < 0.05. Two-way ANOVA followed by Newman-Keuls post-test when variances were equal and Games-Howell when variances were different.

The WD condition produces body and R-WAT weight loss. The loss of R-WAT observed in the old male rats subjected to WD, guided to study possible alterations in the lipid metabolism for the condition of WD, so the next pass for the research was perform the lipidogram assay, determination of glycerol, and palmitic acid (the main free fatty acid in plasma).

#### Glycemia and lipidogram results

**Figure 1:**
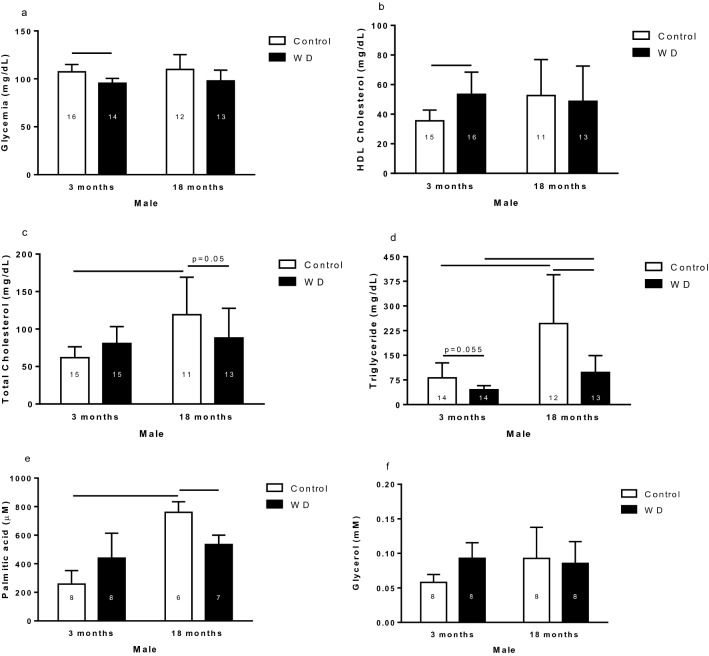
Glycemia (**a**), HDL cholesterol (**b**), total cholesterol (**c**), triglyceride (**d**), palmitic acid (**e**), and glycerol (**f**) of 3- and 18-month-old male control and rats with water deprivation (WD). Data are presented as means (SD), p < 0.05 among the indicated groups. Two-way ANOVA followed by Newman-Keuls or Duncan post-test when variances were equal, and Games-Howell when variances were different. The n for each group is inside the column.

Glycemia (blood glucose concentration) in the 3-month-old males submitted to WD was lower than the respective control (Fig. 1a). The high-density lipoprotein (HDL) cholesterol plasma concentration was higher in the 3-month-old WD males than their respective control (Fig. 1b). Total cholesterol plasma concentration was higher in 18-month-old control males than 3-month-old control males and 18-month-old WD males (Fig. 1c). The TG plasma concentration was higher in control rats than WD rats, and in 18-month-old rats than 3-month-old rats (Fig. 1d). Palmitic acid plasma concentration was higher in 18-month-old control males than 3-month-old control males and 18-month-old WD males (Fig. 1e). The males did not exhibit differences in the glycerol levels (Fig. 1f).

Age and WD condition change energetic metabolism of male rats. The alterations in the metabolism of lipids observed in the WD and old males, led us to question if these results were products of hormone alterations, so the concentration plasma hormones, that participate in the energetic metabolism control, were determined.

#### Hormone levels in the blood

**Figure 2:**
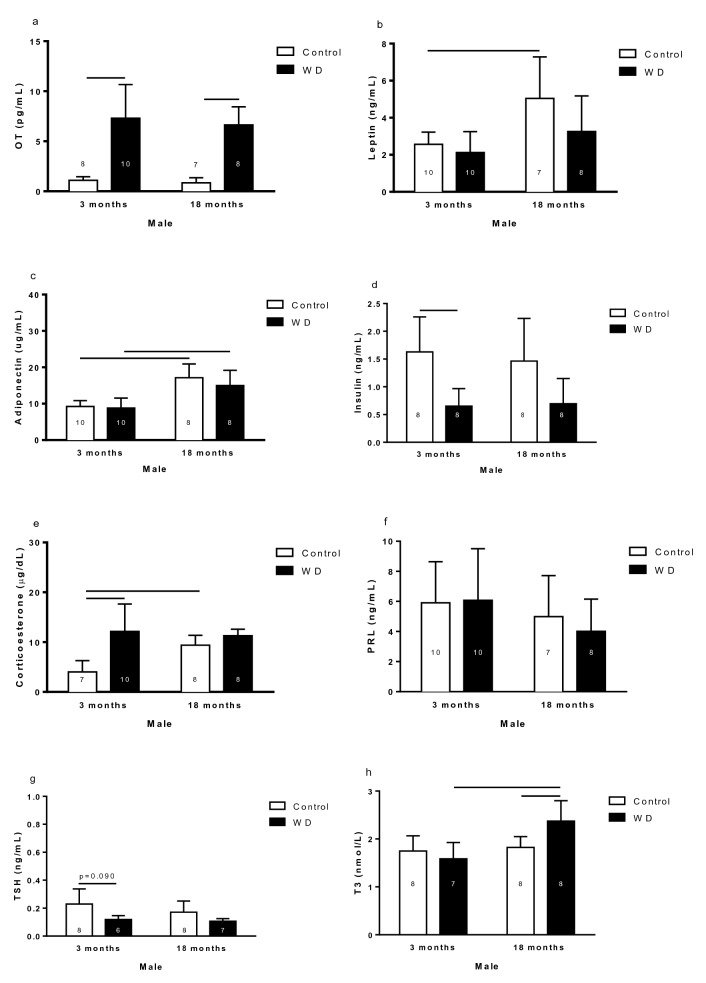
Oxytocin (**a**), leptin (**b**), adiponectin (**c**), insulin (**d**), corticosterone (**e**), prolactin (**f**), thyroid stimulating hormone (TSH) (**g**), and triiodothyronine (**h**) plasma concentration of 3- and 18-month-old male control and rats with water deprivation (WD). Data are presented as means (SD), p < 0.05 among the indicated groups. Two-way ANOVA followed by Newman-Keuls post-test when variances were equal, and Games-Howell when variances were different. The n for each group is inside or above the column.

The OT plasma level was higher in WD males than the control males (Fig. 2a). Leptin plasma levels were higher in 18-month-old control rats than 3-month-old control rats (Fig. 2b). Adiponectin plasma concentration in 18-month-old males was higher than in the 3-month-old males (Fig. 2c). Insulin plasma concentration was lower in 3-month-old WD rats than respective control (Fig. 2d).

Corticosterone (CORT) plasma level was lower in 3-month-old control rats than 3-month-old WD rats and 18-month-old control rats (Fig. 2e). No differences were observed in prolactin (PRL) plasma concentration in male rats (Fig. 2f). The plasma concentration of thyroid stimulating hormone (TSH) was higher in 3-month-old control males than in 3-month-old WD males (Fig. 2g). The plasma levels of triiodothyronine (T3) were higher in 18-month-old WD rats than respective control and 3-month-old WD rats (Fig. 2h).

In male rats, the hormones that regulate energy metabolism and lipid metabolism suffer alterations by WD condition and aging.

#### Relative gene expression

It was decided to determine the expression of *Oxtr* mRNA, to determine OT participation in the alteration of adipose tissue metabolism, and *P-para mRNA*, to determine if this receptor was activated under the conditions established in the study.

**Figure 3:**
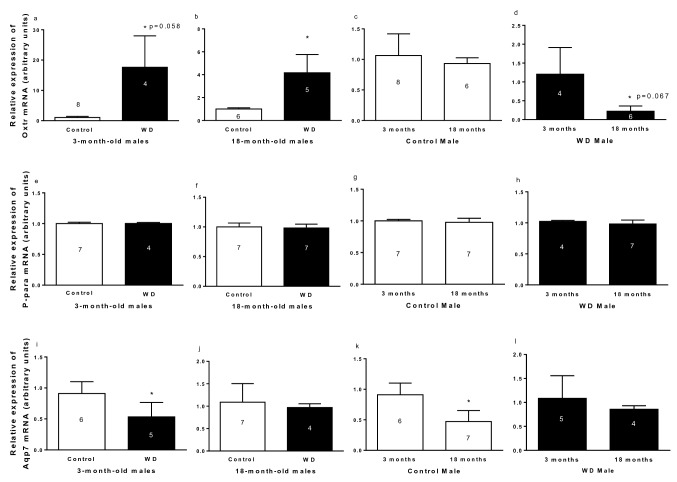
Relative expression of *Oxtr* (**a**–**d**), *Ppara* (**e**–**h**)*, and Aqp7* (**i**–**l**) mRNA in R-WAT of 3- and 18-month-old control and with water deprivation (WD) male rats. Comparison by hydration condition (**a** and **b**; **e** and **f**; **i** and **j**), age (**c** and **d**; **g** and **h**; **k** and **l**). Data are presented as means (SD), *p < 0.05 with respect to control (**a**, **b**, and **i**), 3-month-old (**d** and **k**). Student’s unpaired t-test. The n for each group is inside or above the column.

Relative expression of oxytocin receptor, P-para and Aqp7 mRNA in R-WAT in male is presented in Fig. 3. Rats submitted to WD showed higher expression of *Oxtr* mRNA (Fig. 3a,b). Aging altered the *Oxtr* mRNA expression in WD males, that showed lower *Oxtr* mRNA expression than 3-month-old WD males (Fig. 3d). No changes were observed in the *Oxtr* mRNA expression by aging in control males (Fig. 3c).

No changes were observed in peroxisome proliferator-activated receptor alpha (*P-para)* mRNA expression in R-WAT under conditions established in the study, as is showed in Fig. 3e–h.

The 3-month-old males submitted to WD showed lower *Aqp7* mRNA expression than respective controls (Fig. 3i). No difference in *Aqp7* mRNA expression in R-WAT was observed in 18-month-old control rats (Fig. 3j). Aging, in control group, affected the *Aqp7* mRNA expression, where 18-month-old controls showed lower expression of *Aqp7* mRNA. However, no changes were found by aging in WD rats (Fig. 3k,l).

The increased in the *Oxtr* mRNA expression in animals submitted to WD may indicate a greater participation of OT in the lipid metabolism in the dehydration condition. On the other hand, while age and WD factors decreasing the relative expression of *Aqp7* mRNA in the R-WAT, the P-para mRNA expression is not altered by any of these factors.

### Females

#### Body and R-WAT weight

The body weight of the 18-month-old rats was higher than 3-month-old rats. In addition, 3-month-old female rats with WD showed lower body weight than respective control. Also, the body weight change was bigger in females submitted to WD than respective control group. Furthermore, R-WAT weight of females was higher in the 18-month-old submitted to WD than the respective 3-month-old (Table 1).

The WD produces body weight loss without altering R-WAT weight. Although no loss of R-WAT was observed, the loss of body weight conducted to study possible alterations in the lipid metabolism for the condition of WD, for this, it was performing the lipidogram assay and the determination of glycerol and palmitic acid.

#### Glycemia and lipidogram results

**Figure 4:**
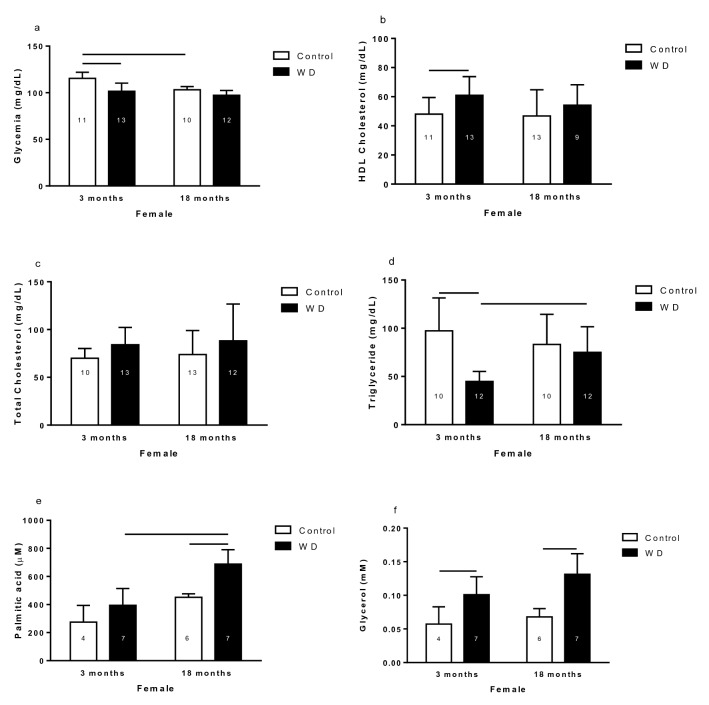
Glycemia (**a**), HDL cholesterol (**b**), total cholesterol (**c**), triglyceride (**d**), palmitic acid (**e**), and glycerol (**f**) of 3- and 18-month-old female control and rats with water deprivation (WD). Data are presented as means (SD), p < 0.05 among the indicated groups. Two-way ANOVA followed by Newman-Keuls or Duncan post-test when variances were equal, and Games-Howell when variances were different. The n for each group is inside the column.

The 3-month-old control female rats exhibited higher glycemia than 3-month-old WD and 18-month-old control female rats (Fig. 4a). The HDL cholesterol plasma concentration was higher in the 3-month-old WD females than their respective control (Fig. 4b). No differences were observed in total cholesterol of female rats (Fig. 4c). The 3-month-old WD rats had lower TG plasma concentration than 3-month-old control rats and 18-month-old WD rats (Fig. 4d). The 18-month-old WD females showed higher palmitic acid plasma concentration than 3-month-old WD females and 18-month-old control females (Fig. 4e). Rats submitted to WD showed higher glycerol plasma concentration than control rats in both 3-month-old and 18-month-old groups (Fig. 4f).

The WD female rats, despite not having an alteration in the R-WAT, they showed a lipolytic state because they had high values of plasma glycerol and palmitic acid, so it was important to determine the plasma concentrations of the hormones that participate in the control of fat metabolism. Also, as the WD female rats presented increased plasma glycerol concentration, it was decided to evaluate the relative expression of *P-para and AQP7 mRNA* in R-WAT.

#### Hormone levels in the blood

**Figure 5:**
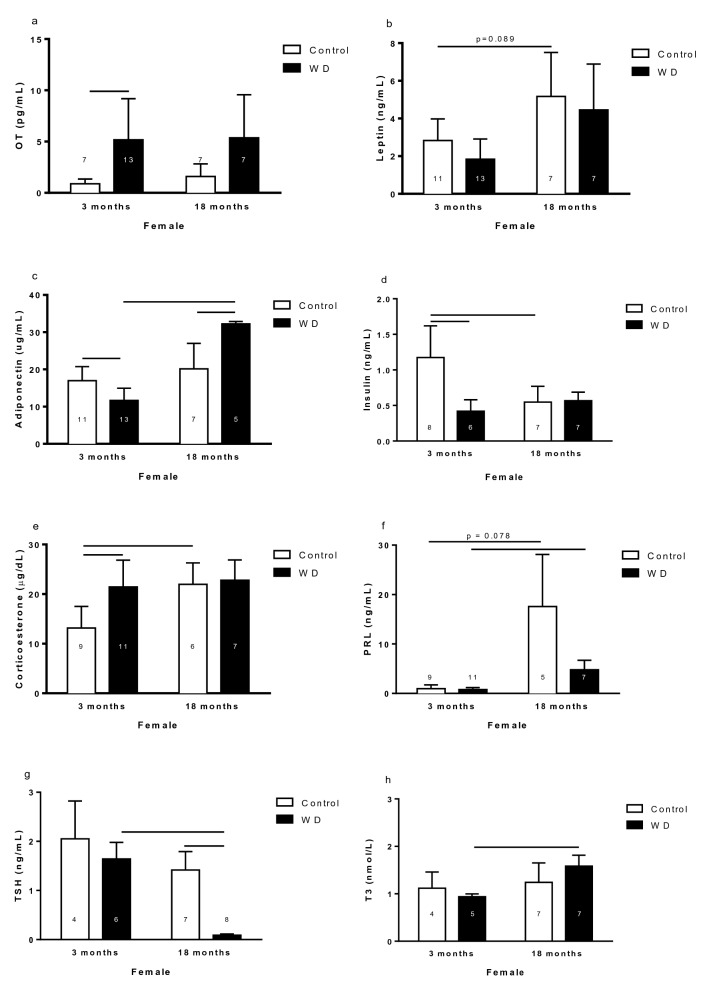
Oxytocin (**a**), leptin (**b**), adiponectin (**c**), insulin (**d**), corticosterone (**e**), prolactin (**f**), thyroid stimulating hormone (TSH) (**g**), and triiodothyronine (**h**) plasma concentration of 3- and 18-month-old female control and rats with water deprivation (WD). Data are presented as means (SD), p < 0.05 among the indicated groups. Two-way ANOVA followed by Newman-Keuls post-test when variances were equal, and Games-Howell when variances were different. The n for each group is inside or above the column.

The OT plasma level was higher in 3-month-old WD females than respective control (Fig. 5a). Leptin plasma levels were higher in 18-month-old control rats than 3-month-old control rats (Fig. 5b). Adiponectin plasma concentration was higher in the 3-month-old controls than the 3-month-old WD, and it was higher in 18-month-old WD females than 18-month-old control females and 3-month-old WD females (Fig. 5c).

The 3-month-old control rats showed higher insulin plasma level than 3-month-old WD rats and 18-month-old control rats (Fig. 5d). CORT plasma level was lower in 3-month-old control animal than 3-month-old WD animals and 18-month-old control animals (Fig. 5e). The PRL plasma concentration was higher in 18-month-old females than 3-month-old females (Fig. 5f). The 18-month-old WD females exhibited lower TSH plasma concentration than their control respective and 3-month-old WD females (Fig. 5g). The 18-month-old WD females showed higher T3 plasma levels than 3-month-old WD females (Fig. 5h).

Hormones that participate in the control of lipid metabolism are altered by age and WD condition in female rats.

#### Relative gene expression

**Figure 6:**
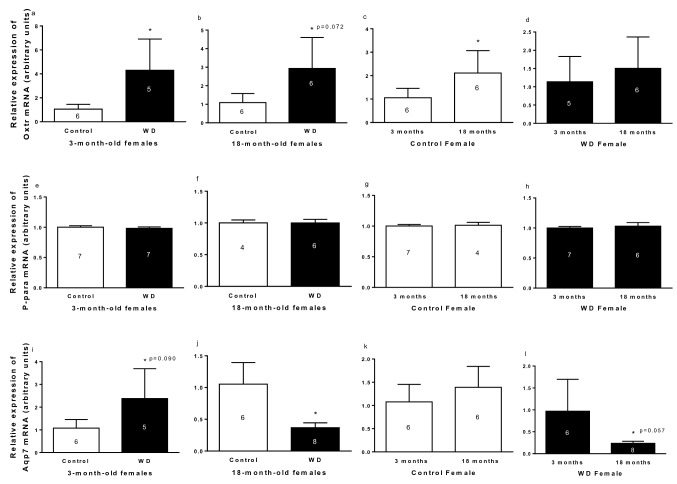
Relative expression of *Oxtr* (**a**–**d**), *Ppara* (**e**–**h**)*, and Aqp7* (**i**–**l**) mRNA in R-WAT of 3- and 18-month-old control and with water deprivation (WD) female rats. Comparison by hydration condition (**a** and **b**; **e** and **f**; **i** and **j**), age (**c** and **d**; **g** and **h**; **k** and **l**). Data are presented as means (SD), *p < 0.05 with respect to control (**a**, **b**, **i**, and **j**), 3-month-old (**c** and **l**). Student’s unpaired t-test. The n for each group is inside or above the column.

Relative expression of oxytocin receptor, P-para and Aqp7 mRNA in R-WAT in male is presented in Fig. 6. Rats submitted to WD showed higher expression of *Oxtr* mRNA (Fig. 6a,b). The 18-month-old control females had higher *Oxtr* mRNA expression than 3-month-old control females (Fig. 6c). No changes were observed by aging in WD females (Fig. 6d).

The *P-para* mRNA expression in R-WAT did not show changes under conditions established in the study, as is showed in Fig. 6e–h.

Hydric condition affected the *Aqp7* mRNA expression in female rats of different forms, according to age. Thus, the 3-month-old females submitted to WD had higher *Aqp7* mRNA expression than 3-month-old control females (Fig. 6i). However, 18-month-old rats submitted to WD showed lower *Aqp7* mRNA expression than their respective controls (Fig. 6j). Aging affected the *Aqp7* mRNA expression in WD group, where 18-month-old females showed lower expression of *Aqp7* mRNA than respective 3-month-old rats (Fig. 6l). However, no changes were found by aging in control female rats (Fig. 6k).

WD increases *Oxtr* mRNA expression, alters the *Aqp7* mRNA expression, and not change the *P-para* mRNA expression.

### Sex differences on WD groups

#### Glycemia and lipidogram results

**Figure 7:**
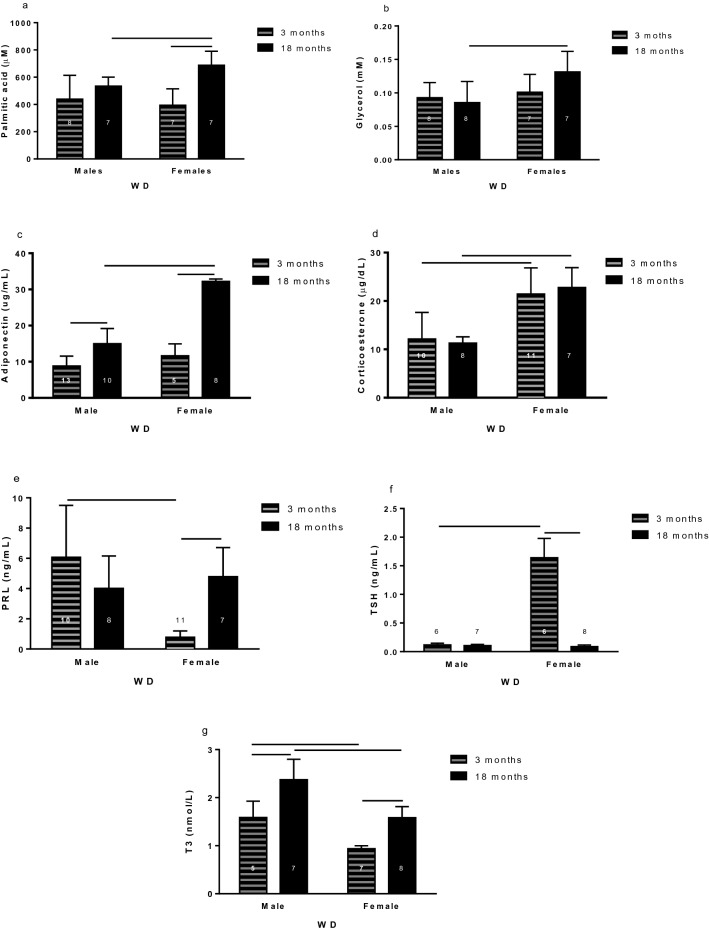
Sex comparation of 3- and 18-month-old rats with water deprivation (WD) of palmitic acid (**a**) and glycerol (**b**), adiponectin (**c**), corticosterone (**d**), prolactin (**e**) thyroid stimulating hormone (TSH) (**f**), and triiodothyronine (**g**) plasma concentration. Data are presented as means (SD), p < 0.05 among the indicated groups. Two-way ANOVA followed by Newman-Keuls post-test when variances were equal, and Games-Howell when variances were different. The n for each group inside or above the column.

When compared by sex, 18-month-old females displayed higher palmitic acid and glycerol plasma concentration than 18-month-old males (Fig. 7a,b).

The sex is a factor that alters lipid metabolism in old rats subjected to WD.

#### Hormone levels in the blood

The 18-month-old females exhibited adiponectin plasma level higher than 18-month-old males (Fig. 7c). The 3- and 18-month-old females showed higher CORT plasma concentration than respective males (Fig. 7d). The 3-month-old males showed higher PRL plasma levels than 3-month-old females (Fig. 7e). The 3-month-old females showed higher TSH plasma concentration than 3-month-old males (Fig. 7f). Males had higher T3 plasma levels than females (Fig. 7g).

In response to the WD condition, the hormones that participate in the control of energy metabolism vary according to the sex of the animal.

#### Relative gene expression

**Figure 8:**
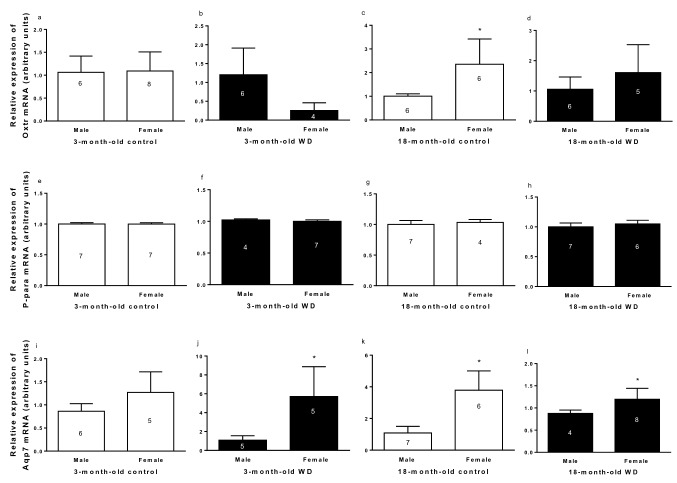
Relative expression of *Oxtr* (**a**–**d**), *Ppara* (**e**–**h**)*, and Aqp7* (**i**–**l**) mRNA in R-WAT of 3- and 18-month-old control and male and female rats with water deprivation (WD). Comparing by sex. Data are presented as means (SD), *p < 0.05 with respect to male. Student’s unpaired t-test. The n for each group is inside or above the column.

No differences according to sex were observed in 3-month-old rats and 18-month-old WD rats (Fig. 8a,b,d). However, sex comparison found that 18-month-old control females had higher *Oxtr* mRNA expression than respective males (Fig. 8c).

No changes were observed in *P-para* mRNA expression in R-WAT under conditions established in the study, as is showed in Fig. 8e–h.

Sex comparison indicated that females had higher *Aqp7* mRNA expression than males in the 3-month-old WD groups, 18-month-old control groups, and 18-month-old WD groups (Fig. 8j–l). No difference was observed in *Aqp7* mRNA expression according to sex in 3-month-old control groups (Fig. 8i).

In the WD condition, sex *affects the* relative expression of *Aqp7* mRNA, while the relative expression of *Oxtr* and *P-para* mRNA are not modified.

Age and sex alter lipid metabolism, the hormones that regulate it, and the relative gene expression of some R-WAT genes in Wistar rats submitted to WD.

## Discussion

Alteration in energy metabolism produced by WD varies according to sex and aging. The current study assessed body and retroperitoneal white adipose tissue (R-WAT) weight, glucose and lipid metabolism, hormone secretion, and relative gene expression of the R-WAT at different ages of male and female rats submitted to WD.

The data presented here indicate that WD produces weight loss in animals, and this loss may be partly the result of the loss of body fluid and the reduction of adipose tissue thanks to lipolysis^14,15^. The lack of change in the weight of R-WAT of 18-month-old WD females could indicate that lipolysis was happening in the subcutaneous adipose tissue; however, in old WD male rats, lipolysis happens mainly in visceral adipose tissue^11,16^. The lipolysis observed in old WD animals may have been stimulated by OT, since it has been shown that OT increases lipolysis of adipose tissue and β-oxidation, reduces visceral fat and body weight in obese animals induced by diet, and in dehydrated female rats produces an increase in the plasma concentration of free fatty acids^5–7,17^. In addition to OT, T3 by direct action on adipose tissue could be participating in lipolysis stimulation in 18-month-old males^18^.

In addition, adult male rats subjected to WD had decreased TG in serum, possibly as a result of intracellular accumulation of TG in the liver, by stimulation of corticosterone^19–21^. In the case of old WD males, the decrease of TG and total cholesterol in serum may be due to the increase in plasma T3 in these animals^18,22^. The increase in serum HDL cholesterol observed in 3-month-old WD animals could be due, to the effect of CORT in the liver^23^. On the other hand, the lack of effect of CORT in old animals may be because old animals have elevated CORT level even in the condition of normohydration, which causes the receptors for them in different tissues to be reduced, as has already been demonstrated in different parts of the brain of old male rats^24^.

On the other hand, in the 3-month-old WD animals, the blood glucose values decreased in the same way as the plasma insulin concentration, but the old animals in WD condition exhibited no change in these parameters, showing that the maintained glycemia in these animals was possibly happening through the gluconeogenesis, mediated by decreased insulin and increased glucagon^19,21^.

Plasma TSH, leptin and adiponectin varies with age, sex and, according to our results, water status^25^. Thus, testosterone and estradiol increase the plasma TSH concentration in adults rats, so the decrease in this hormone observed in 3-month-old WD rats may have been due to the decrease in the testosterone concentration in the plasma of these animals and, in the case of 3-month-old females, having a higher concentration of estradiol than older rats^26,27^. The decrease in TSH in 18-month-old WD females could be related to increase in adiponectin in this group, since a negative correlation was observed in these hormones, as observed in our results^28^.

Interesting, the expression of *P-para* mRNA in R-WAT did not present any alteration in animals submitted to dehydration. However, the expression of *Aqp7* mRNA in R-WAT is altered by WD, age, and sex. Although, it is know that estrogen induces the expression of Aqp7, the differences found in the expression of *Aqp7* mRNA according to hydric condition and aging, require further research to decipher the mechanisms that led to these results, since these differences have not been previously shown^29^.

In conclusion, our results show that WD and aging alter the energy metabolism of rat adipose tissue. In addition, sex is a very important factor to consider since significant differences were observed according to sex of the animal. This is an area that still requires extensive research because it has more new mechanisms to be deciphered (Supplementary Information).

## Material and methods

### Animals

Wistar rats were obtained from the animal facility located at the Campus-USP of Ribeirão Preto, University of São Paulo, Brazil. Male and female rats were subjected to experiments at 3 and 18 months old. They were maintained under controlled temperature of 23 ± 2 °C and exposed to a 12:12 h light–dark cycle (light period: 06–18 h) and tap water and standard diet (QuimtiaNuvilab®—3.86 kcal/g being 4% lipids, 22% proteins, and 60% carbohydrates). Male rats were housed in groups of 5 until that they reached the body weight of 500 g, after that, they were housed in pairs, females were kept in groups of 4. Each animal was identified with a number on the tail. Experiments were performed in the morning from 08:00 until 11:00 h. Animals that show a tumor, excessive weight loss, infection, and some sign of pain or health problems were excluded from the experiment (5 female and 2 male 18 months old). This research was conducted according to the “Guide for the Care and Use of Laboratory Animals” (NIH; Publication No. 85-23, revised 1996), and the assay procedures were approved by the Ethical Committee for Animal User of the School of Medicine of Ribeirao Preto, University of Sao Paulo (protocol # 014/2014-1). Blinding was used during the conduct of the experiment.

We infer that 18 month in the life of rats corresponds to sixties in human life, since our Wistar rat’s colony have life spans of about 2 years that correlate with 80 years of human life, and at 18 months, the female rats were in estropause (reproductive senescence).

### Blood and tissue collection

Male and female rats at 3 and 18 months old were submitted or not to water deprivation (WD) for 48 h, assigned by a simple randomization method. Before and after of the period of WD, body weight was determined, and after being weighed, they were euthanized by decapitation, without use of anesthetic agent to avoid the effects of this to metabolism and endocrine system^30–33^. Blood was gathered from trunk in refrigerated tubes containing or not heparin (10 μL/mL of blood) to obtain plasma and serum, respectively, and a drop of blood was used to determinate glycemia using a glucometer and reactive strips for glucometer (Accu-Check Performa).

The estrous cycle of the female rats was determined by vaginal smear and the plasma of those rats in diestrus were used for glycemia, lipidogram, and hormonal measurements.

Retroperitoneal white adipose tissue (R-WAT) was removed and weighed. R-WAT was stored at − 70 °C until the day of the mRNA extraction.

### Lipidogram assay

The measures of triglycerides (TG), high-density lipoprotein (HDL) cholesterol, and total cholesterol were performed following the guidelines of the commercial kit manufacturer [Triglycerides Liquiform (Ref. 87), HDL Cholesterol (Ref. 13), Liquiform Cholesterol (Ref. 76), Labtest Diagnóstica, SA, Brazil]. All quantifications were made in triplicate. Free fatty acid quantification was made by commercial colorimetric kit [EnzyChromTM Free Fatty Acid Assay (EFFA-100), BioAssay Systems] and the glycerol by commercial colorimetric kit [Glycerol Assay Kit (MAK117), Sigma-Aldrich-^®^], following manufacturer's specifications.

We decided to investigate concentration of palmitic acid and glycerol to determinate the effect of water deprivation in the lipolysis of retroperitoneal adipose tissue.

### Hormonal quantification

Commercial ELISA kits were used to quantify insulin (Alpco, Salem, NH, USA), leptin and adiponectin (EMD Millipore Corporation, Billerica, MA, USA), thyroid stimulating hormone (TSH) (Crystal Chem, Grove Village, IL, USA), and T3 (MyBioSource, San Diego, CA, USA). Hormonal quantification was performed in duplicate.

OT were withdrawed from 1 mL of plasma with acetone and petroleum ether, whereas corticosterone (CORT) was obtained from 25 μL of plasma with 1 mL of ethanol. OT and CORT measurements were performed using radioimmunoassay (RIA) technique described by Haanwinckel et al. and Vecsei^34,35^. Measurements were performed in duplicate in the same assay. The sensitivity of the analyze and the intra-assay coefficient of variation were respectively 0.1 pg/mL and 4.1% for OT and 7.8 μg/dL and 3.2% for CORT.

The plasma prolactin (PRL) level was measured by RIA method, where the antibody used was supplied by the National Hormone and Peptide Program (Harbor-UCLA Medical Center, CA, USA). The lower limit of detection was 0.10 ng/mL, and the intra-assay coefficient of variation was 2.5%.

The adiponectin and leptin hormones were studied to determinate if WD changes their plasma concentrations due to alteration of adipose tissue. Prolactin, TSH and T3 were studied, because they stimulate the lipid metabolism, so we would like to know if WD altered the plasma concentration of them and these could be a possible explanation of alteration of metabolism lipids, thinking more in an organism working with all their system.

### RNA isolation and semi-quantitative real-time PCR (QT-PCR)

Total RNA was extracted using 0.1 g R-WAT and Trizol reagent (Invitrogen), according to the manufacturer’s instructions. Quantitation and purity of the mRNA were corroborated in a spectrophotometer (SpectraMax^®^ i3x Multi-Mode Microplate Reader). Absorbance ratios of 260/280 and 260/230 nm were used to determine the purity.

The commercial High-Capacity cDNA Reverse Transcription kit (Applied Biosystems^®^) was used to make the complementary DNA synthesis (cDNA) beginning with 500 ng of RNA. The thermocycler (GeneAmp PCR System 9600, Applied Biosystems) was used to make the reverse transcription with the following conditions: 10 min at 25 °C and 120 min at 37 °C, after the samples were kept at − 20 °C.

The QT-PCR was performed in triplicate, using Taqman^®^ assays (Applied Biosystems^®^): *Oxtr* (Rn00563503_m1), *Aqp7* (Rn00569727_m1), *P-para* (Rn00566193_m1), and *Rat ACTB* (actin, beta; Rn00667869_m1) as endogenous control gene, in the 7500 QT-PCR System (Applied Biosystems^®^). The threshold cycle (Ct) was used to calculate the relative expression of the target gene and results were evaluated as stated by the ΔΔCt method.

### Statistical analysis

Sample size was estimate assuming α = 5% and power of 0.80 from previous study (G*Power, Düsseldorf). Results are presented as the means and Standard Deviation (SD). The Statistica (StatSoft, USA) and SPSS Statist (IBM, USA) software were used to analyze the data. Body weight change, R-WAT weight, glycemia, lipidogram parameters, and plasma concentration hormones were analyzed by two-way ANOVA followed by Newman-Keuls or Duncan post-test when variances were equal, Games-Howell when variances were different. To analyze the mRNA relative expression, Student’s unpaired t-test was used. It was set a p < 0.05 (two-tailed) as significance level. The “n” was employed to represent the number of animals used.

### ARRIVE guidelines

We confirm that the manuscript follows the recommendations in the Arrive guidelines.

## Supplementary Information

Supplementary Information.

## Data Availability

The datasets generated during the current study are available from the corresponding author on reasonable request.
