# Supplementary material for: Age and sex influence the response in lipid metabolism of dehydrated Wistar rats
Source: Sci Rep. 2022 Jun 2;12:9164. doi: 10.1038/s41598-022-11587-w (PMC9163080; doi:10.1038/s41598-022-11587-w)
Supplement: Supplementary file 1 — Supplementary Information. [file 41598_2022_11587_MOESM1_ESM.pptx]

## Slide 1
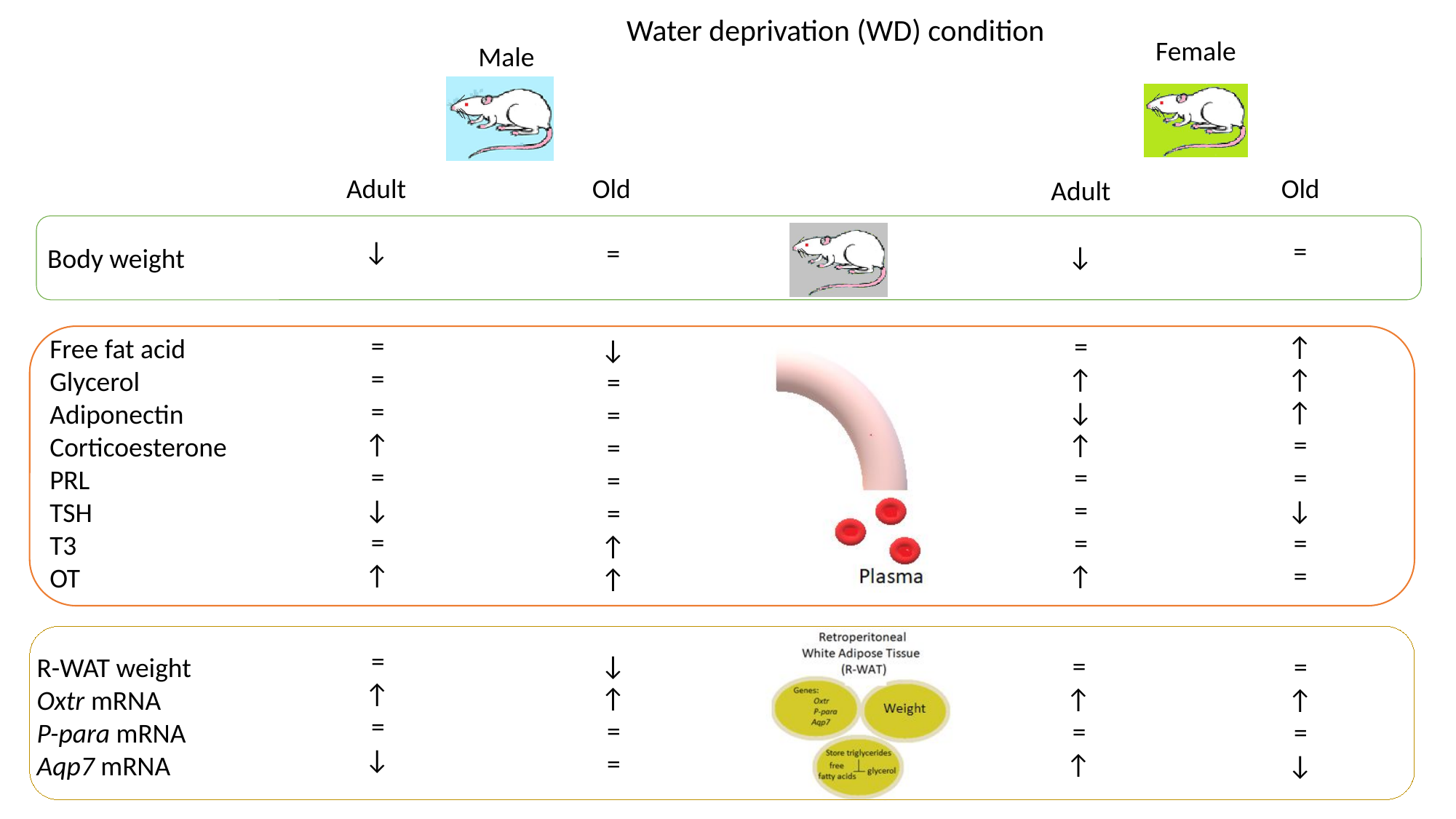

Water deprivation (WD) condition
Female
Male
Adult
Old
Old
Adult
=
↓
=
↓
Body weight
 =
 =
 =
↑
 =
↓
 =
↑
↑
↑
↑
 =
 =
↓
 =
 =
 =
↑
↓
↑
 =
 =
 =
↑
Free fat acid
Glycerol
Adiponectin
Corticoesterone
PRL
TSH
T3
OT
↓
 =
 =
 =
 =
 =
↑
↑
 =
↑
 =
↓
↓
↑
 =
 =
 =
↑
 =
↑
 =
↑
 =
↓
R-WAT weight
Oxtr mRNA
P-para mRNA
Aqp7 mRNA
